# Expression of Proinflammatory and Regulatory Cytokines via NF-*κ*B and MAPK-Dependent and IFN Regulatory Factor-3-Independent Mechanisms in Human Primary Monocytes Infected by *Mycobacterium tuberculosis*

**DOI:** 10.1155/2011/841346

**Published:** 2010-12-21

**Authors:** Elena Giacomini, Maria Elena Remoli, Marta Scandurra, Valérie Gafa, Manuela Pardini, Lanfranco Fattorini, Eliana M. Coccia

**Affiliations:** ^1^Dipartimento di Malattie Infettive, Parassitarie ed Immmuno-mediate, Istituto Superiore di Sanità, I-00161, Rome, Italy; ^2^EA4303 “Inflammation et Immunité de l'épithélium Respiratoire”, IFR53, UFR de Pharmacie, Université de Reims Champagne-Ardenne, 51096 Reims, France

## Abstract

Knowledge of the molecular events regulating the innate response to *Mycobacterium tuberculosis* (Mtb) is critical for understanding immunological pathogenesis and protection from tuberculosis. To this aim, the regulation and the expression of regulatory and proinflammatory cytokines were investigated in human primary monocytes upon Mtb infection. We found that Mtb-infected monocytes preferentially express a proinflammatory cytokine profile, including IL-6, TNF-*α*, and IL-1*β*. Conversely, among the regulatory cytokines, Mtb elicited IL-10 and IL-23 release while no expression of IL-12p70, IL-27, and IFN-*β* was observed. The analysis of the signalling pathways leading to this selective cytokine expression showed that in monocytes Mtb activates MAPK and NF-*κ*B but is unable to stimulate IRF-3 phosphorylation, a transcription factor required for IL-12p35 and IFN-*β* gene expression. Thus, by inducing a specific cytokine profile, Mtb can influence the immunoregulatory properties of monocytes, which represent important target of novel vaccinal strategies against Mtb infection.

## 1. Introduction

Mortality and morbidity caused by the pulmonary pathogen *Mycobacterium tuberculosis* (Mtb) remain alarmingly high [1]. Although much is known about the immunology of tuberculosis (TB), the precise nature of the protective immune mechanisms against Mtb has not been completely defined. Indeed, it has been estimated that 30% of the exposed individuals will become tuberculin-positive patients and, among the infected individuals, only 5%–10% will develop clinical manifestations of active TB, while the majority controls the infection but not completely eradicates the bacteria developing into a latent TB [2]. Therefore, the characterization and comprehension of the immune mechanisms leading to the control of initial infection and to the prevention of reactivation of latent infection are crucial to contain this disease. A complex series of interactions among various cell populations is involved in the containment of Mtb infection [2]. In particular, macrophages and dendritic cells (DCs) are essential players against Mtb given their involvement in phagocytosis, antigen processing and presentation, and cytokine production [3, 4]. After the interaction with Mtb in the alveoli, macrophages and DC become activated and participate to the immune response against Mtb infection playing different roles [5]. DCs are engaged in inducing T cells in virtue of their production of Th1/IFN-*γ*-inducing cytokines and expression of costimulatory molecules, while macrophages are primarily involved in the formation of the granuloma, where tissue macrophages harboring tubercle bacilli are surrounded by and interact with natural killer (NK) and effector T lymphocytes. Indeed, NK and T cells are recruited after Mtb infection and produce IFN-*γ* and TNF-*α*, which play a central role in the host defense against Mtb given their capacity to activate antimicrobial mechanisms in monocytes and macrophages [6]. Besides macrophages and DC, circulating monocytes are increasingly implicated in defense against a range of microbial pathogens by supplying tissues with macrophage and DC precursors [7]. We have identified new mechanisms of Mtb immune evasion that relies on the capacity of Mtb to interfere with monocyte differentiation into DC [8, 9]. In particular, our studies provided evidences that CR3 engagement on monocytes by mycobacteria leads to p38 mitogen-activated protein kinase (MAPK) and ATF-2 phosphorylation and that antibody-dependent CR3 blockade or treatment with a specific p38 MAPK inhibitor caused a notable increase in CD1 molecule expression in DC derived from mycobacteria-infected cells [9]. In addition, more recently the involvement of p38 MAPK and Signal Transducer and Activator of transcription pathways has been described in the expression of Interleukin-10 (IL-10), which inhibits the differentiation of bystander noninfected monocytes into DC [8]. Thus, by limiting the generation of functionally active DC, Mtb could block the expansion of T lymphocytes lacking effector function and, in turn, reducing the T cell help provided to infected alveolar macrophages for killing the intracellular pathogens.

To further investigate the response of monocyte to Mtb infection, we sought to investigate the profile of proinflammatory and regulatory cytokines and the molecular mechanisms underlying this expression. Our results indicate that human monocytes produce preferentially proinflammatory cytokine profile, including IL-6, TNF-*α*, IL-1*β*. Interestingly, we found that although monocytes activated IFN regulatory factor (IRF)-3 and synthesized IFN-*β* in response to LPS, they failed to do this upon Mtb challenge. Accordingly, the IFN-*β*-mediated expression of IL-27p28 did not occur in Mtb-infected monocytes as well as the release IL-12p70, a cytokine partially dependent on IRF-3 activation. Conversely, the robust production of IL-23, another IL-12 family member, was regulated by MAPK pathway. Collectively, our results highlight the capacity of Mtb to stimulate in monocytes a specific cytokine profile whose regulation was mainly dependent on MAPK and NF-*κ*B signalling.

## 2. Materials and Methods

### 2.1. Reagents

LPS from Escherichia coli 0111:B4 (Sigma-Aldrich, St. Louis, MO, USA) was used at 1 *μ*g/ml. The p38 MAPK inhibitor SB203580 and the ERK inhibitor PD98059 (Calbiochem Biochemicals, San Diego, CA, USA), at an optimal concentration of 3 *μ*M, were added 30 min before Mtb infection.

### 2.2. Bacterial Strains, Media, and Growth Conditions

Mtb H37Rv (ATCC 27294) was grown and prepared as previously described [5]. Mtb was harvested at midlogarithmic growth phase. Bacterial viability was determined by counting the number of colony-forming units (CFUs) on Middlebrook 7H10 agar plates. All bacteria preparations were analyzed for LPS contamination by the Lymulus lysate assay (BioWhittaker, Verviers, Belgium) and contained less than 1 EU/ml.

### 2.3. Monocyte Preparation and Infection

Monocytes were prepared as previously described [5]. Monocytes were cultured at 1 × 10^6^ cells/ml in RPMI 1640 (BioWhittaker) supplemented with 2 mM L-glutamine and 15% FCS (BioWhittaker). No antibiotics were added to the cultures. Monocytes were infected with a multiplicity of infection (MOI) of one bacterium/cell, which was previously found to be optimal for the infection of primary cells [5]. Mtb preparations were sonicated prior to infection of monocyte cultures. The infection was evaluated at various time points (0, 4, and 24 hr) by CFU assay to determine the number of intracellular bacteria, as previously described [10]. Briefly, 2 hr after infection, the cell cultures were gently washed (three times) with RPMI 1640 and centrifuged at 150 × g for 10 min to selectively spin down cells while extracellular bacteria remain in the supernatants. Cells were resuspended in complete medium and cultured for the times indicated. T0 refers to cell cultures washed after 2 hr of infection, where a 20% reduction of the original MOI was observed. However, we found that Mtb was able to infect and to survive inside the monocytes as the number of CFU remained constant over a 24-hr period of examination (data not shown).

### 2.4. Cytokine Determinations

Supernatants from monocyte cultures were harvested 24 hr after infection or treatments, filtered by 0.2 *μ*m low protein-binding syringe filters (Millex-GV, Millipore, Bedford, MA, USA), and stored at −80°C. IL-6, IL-10, IL-12 p70, TNF-*α*, and IL-1*β* were measured with the human inflammation cytometric bead assay (CBA) (BD Bioscience, Pharmingen, San Diego, CA, USA). The sensitivity of this assay varies from 1.9 pg/ml for IL-12p70 to 7.2 pg/ml for IL-1*β*. IL-23 was assayed with ELISA kit (Bender MedSydtem, Burlingame, CA, USA). The sensitivity is 20 pg/ml for IL-23. All assays were conducted according to manufacturers' instructions. 

### 2.5. RNA Isolation and Real-Time PCR Quantification

RNA was extracted from DC with RNeasy kit (Qiagen Inc., Valencia, CA, USA) according to the manufacturer's instructions. A phenol/chloroform extraction was performed to inactivate residual mycobacterial particles. Reverse transcriptions were performed as previously described [11]. Quantitative PCR assays were performed in triplicate using the Platinum Taq DNA Polymerase (Invitrogen Life Technologies Frederick, MD) and the SYBR Green I (BioWittaker Molecular Applications, Rockland, ME) on a LightCycler (Roche Diagnostics, Basel, CH). The sequences of primer pairs have been previously described [12, 13]. A calibration curve of a purified positive control RT-PCR product, to whom arbitrary values were assigned, was used to calculate the value of a target gene. The quantification standard curves were obtained using dilutions (4-log range) of the purified positive control RT-PCR product in 10 *μ*g/ml sonicated salmon sperm DNA. Quantification data are presented as a ratio to the GAPDH mRNA level present in the same sample and represent the mean ± SE of triplicate values. Only ratios with a SE 0.2 log (95% confidence limits) were considered for the determination of induction levels. The standard errors (95% confidence limits) were calculated using the Student's *t*-test. 

### 2.6. Western Blot Analysis

Western blot was performed as previously described [13]. Briefly, 30 *μ*g of total cell extracts were separated by SDS-PAGE gel and blotted onto nitrocellulose membranes. Blots were incubated with 1 *μ*g/ml of rabbit polyclonal antibodies against IRF-3 (Santa Cruz Biotechnology, Santa Cruz, CA, USA) and reacted with anti-rabbit HRP-coupled secondary antibody (dilution 1 : 2000; Amersham Pharmacia Biotech, Little Chalfont, UK) using an ECL system. Western blots were performed on 7% SDS-PAGE gel to detect the slower migrating phosphorylated form (IRF-3P), while the IRF-3 content was evaluated in a shorter run on 10% SDS gels.

### 2.7. Electrophoretic Mobility Shift Assay (EMSA)

Synthetic double-stranded oligonucleotides were end labeled with [*γ*-32P]ATP by T4 polynucleotide kinase. Binding reactions mixture (20 *μ*l final volume) contained labeled oligonucleotide probes (30,000 cpm) in binding buffer (4% glycerol, 1 mM MgCl_2_, 0.5 mM EDTA, 0.5 mM DTT, 50 mM NaCl, 10 M Tris-HCl (pH 7.5), and 1 *μ*g poly (dI)-poly(dC)). Nuclear lysates (5 *μ*g) were added, and the reaction mixture was incubated for 30 min at room temperature. For supershift analysis, 1 *μ*g of anti-p65 (Santa Cruz Biotechnology, Santa Cruz, CA, USA) was added to the reaction. Glycerol was added to 13% (v/v), and samples were analyzed on 5% polyacrylamide gels with 0.5x TBE (1x TBE is 50 mM Tris-borate (pH 8.2) and 1 mM EDTA) for 1.5 hr at 200 V at 18°C. The oligonucleotide used to monitor the NF-*κ*B binding to *κ*B sequences within the IFN-*β* promoter was 5′-AGTGGGAAATTCCTCT-3′.

### 2.8. Statistical Analysis

Statistical analysis was performed by a two-tailed Student *t*-test for paired data using Java Applets & Servlets for Biostatistics. A *P*-value <.05 was considered statistically significant. 

## 3. Results and Discussion

### 3.1. Proinflammatory and Regulatory Cytokine Release from Mtb-Infected Monocytes

**Figure 1:**
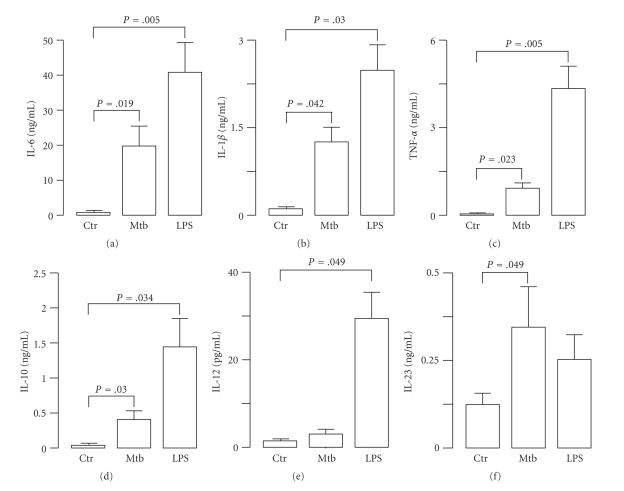
Cytokine production in Mtb-infected or LPS-treated human monocytes. Monocytes were infected with Mtb (MOI = 1) or treated with LPS (1 *μ*g/ml). After 24 hr cell culture supernatants were collected and determination of cytokine content was performed by CBA or ELISA. The results represent the mean ± SE of four separate experiments performed with different donors.

Given the importance of cytokines in orchestrating the immune response against Mtb [3], we sought to investigate how Mtb stimulates the expression profile of proinflammatory and regulatory cytokines in human primary monocytes, whose role in the immune response against pathogens is critical in supporting the development of tissue macrophages and resident DC. To this aim, supernatants were harvested from monocyte cultures infected for 24 hr with Mtb (MOI 1 : 1), and the release of proinflammatory and regulatory cytokines was analyzed (Figure 1). As control, the cultures were stimulated for 24 hr with LPS (1 *μ*g/ml). Monocytes responded to Mtb or LPS stimulation by producing IL-6, TNF-*α*, and IL-1*β* and the regulatory cytokines IL-10 and IL-23, although at different extent. Conversely, the weak secretion of IL-12p70 from LPS-treated monocytes was absent in monocyte cultures infected with Mtb. These results indicated that Mtb differentially induces the release of IL-12p70 and IL-23 by infected monocytes and, in turn, modulates the T cell-dependent inflammatory responses by acting on the balance between Th1 and Th17 cells [14]. Interestingly, our observations are in line with recent findings showing the involvement of IL-23 in mediating protection against intracellular pathogens [15, 16].

### 3.2. Expression and Regulation of IL-12 Family Members in Mtb-Infected Monocytes

**Figure 2:**
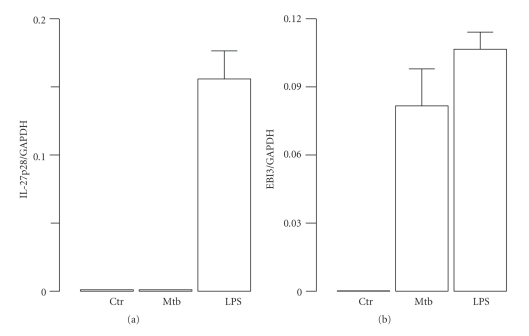
Expression of IL-27 subunits in Mtb-infected or LPS-treated human monocytes. RNA was extracted from monocytes infected with Mtb (MOI = 1) or LPS (1 *μ*g/ml) for 8 hr. Real-time reverse transcription-polymerase chain reaction was performed to measure the expression of IL-27p28 and EBI3 subunits. The results are shown as a ratio to the GAPDH level and represent the mean ± SE of triplicate values. The results shown are from one out of three experiments performed with different donors that yielded similar results.

**Figure 3:**
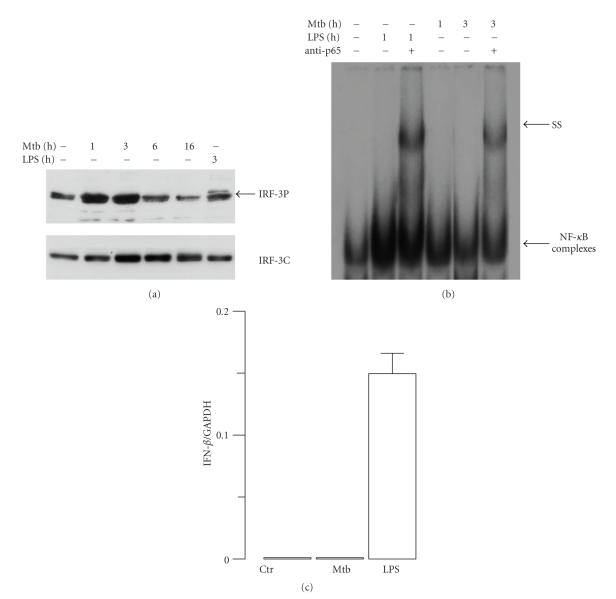
Analysis of IFN-*β* gene regulation in Mtb-infected or LPS-treated human monocytes. (a) Total cell extracts were prepared at different times following infection with Mtb or LPS treatment. Whole cell extracts (30 *μ*g) were analyzed on an SDS-7% PAGE gel and subjected to immunoblot analysis with anti-IRF-3 antibody to detect the phosphorylated IRF-3 isoform (IRF-3P; upper panel). The total content of IRF-3 was evaluated as an internal loading control (IRF-3C; lower panel). The results shown are from one of three experiments performed with cell extracts from different monocyte cultures that yielded similar results. (b) Nuclear extracts were prepared from Mtb-infected at different time points or from cells treated for 1 hr with LPS. Five *μ*g of nuclear proteins were subjected to EMSA analysis using as oligonucleotide the *κ*B-IFN-*β* sequence. Supershift assays were performed after incubation with anti-p65 Abs as indicated (ss). (c) RNA was extracted from monocytes infected with Mtb (MOI = 1) or treated with LPS (1 *μ*g/ml) for 8 hr. Real-time reverse transcription-polymerase chain reaction was performed to measure the expression of IFN-*β*. The results are shown as a ratio to the GAPDH level and represent the mean ± SE of triplicate values. The results shown are from one out of three experiments performed with different donors that yielded similar results.

To further investigate the expression of IL-12 family member in Mtb-infected monocytes, we extended our analysis to another member of IL-12 family, such as IL-27, a heterodimeric protein composed of IL-27p28 and EBI3 [17]. To this aim, total RNA was extracted 8 hr after Mtb infection or LPS treatment, and the transcripts for EBI3 and IL-27p28 subunits were evaluated by real-time RT-PCR (Figure 2). The data showed that LPS induced the transcription of both IL-27 subunits while Mtb promoted only the expression of EBI3 subunit. These findings suggested that signalling pathways regulating the expression of IL-12 and IL-27 mediated by the IRF-3/IFN-*β* axis are not operative in Mtb-infected monocytes. Indeed, evidences indicating the involvement of both NF-*κ*B and IRF-3 in the expression of IL-12p35 have been provided by Goriely and collaborators [18, 19]. In addition, our previous study showed that the expression of IL-27p28 subunit was induced by IFN-*β* alone or during LPS-induced maturation of DC in a type I IFN-dependent manner through IRF-1 activation [12]. Therefore, we sought to investigate the expression of IFN-*β* and the related IRF-3 activation in monocytes challenged with Mtb (Figure 3). IRF-3 is a transcription factor present in the cytoplasm in a latent form, which is activated by two kinases known as IKK-*ε* and TBK-1 at the level of specific serine residues (Ser385 and Ser386) present at the C-terminal [20]. In contrast with previous data showing the ability of Mtb to promote IFN-*β* expression in human DC via NF-*κ*B and IRF-3 activation [13], neither IFN-*β* nor IRF-3 induction was observed in monocytes infected with Mtb in spite of a strong NF-*κ*B binding activity to kB sequences within IFN-*β* promoter (Figure 3). In particular, to investigate IRF-3 activation, immunoblots were performed with whole cell extracts prepared at different time points following infection of monocytes with Mtb or after 3 hr of LPS treatment (Figure 3(a)). To study whether Mtb infection was able to activate NF-*κ*B complex, monocytes were infected with Mtb for 1 or 3 hr or treated with LPS for 1 hr. NF-*κ*B DNA binding was detected 3 hr after Mtb infection and 1 hr after LPS treatment. We confirmed the activation of NF-*κ*B by supershift experiments using Abs raised against the p65 subunit (Figure 3(b)). In addition, the expression of IFN-*β* was investigated by real-time RT-PCR performed on mRNA extracted from Mtb-infected or LPS-treated monocytes (Figure 3(c)). While LPS-treated monocytes expressed IFN-*β* mRNA, Mtb failed to stimulate IFN-*β* transcription at 8 hr after infection (Figure 3(c)) and at later time points (data not shown). Collectively, these data showed that although monocytes possess the machinery to activate IRF-3 in response to LPS (Figure 3), Mtb does not trigger such pathways and, therefore, does not elicit the expression of IL-27 and IL-12p70. The TLR-4-mediated activation of IRF-3, occurring in Mtb-infected DC, seems to be impaired in monocytes through a mechanism not yet identified. We can also envisage that Mtb triggers different receptors on monocytes and DC leading to the activation of distinct intracellular pathways and, in turn, to the secretion of specific cytokine profiles.

### 3.3. Regulation of TNF-*α* and IL-23 Expression via MAPK in Mtb-Infected Monocytes

**Figure 4:**
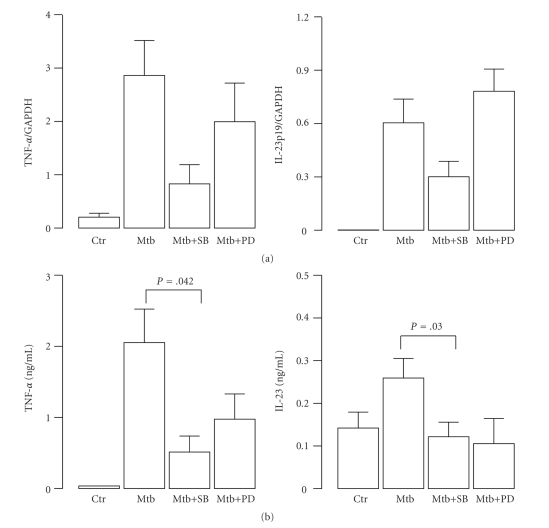
Regulation of IL-23 expression by MAPK in Mtb-infected human monocytes. Monocytes were pretreated or not with p38 (SB203580) or ERK (PD98059) inhibitors for 30 min and afterwards were infected with Mtb as indicated. (a) After 8 hr, RNA was extracted and real-time RT-PCR was performed to measure the expression of TNF-*α* and IL-23p19 subunit transcripts. The results shown are from one out of three experiments performed with different donors that yielded similar results. (b) After 24 hr, the cell culture supernatants were collected, and the production of TNF-*α* and IL-23 was measured by CBA or ELISA assay. The results represent the mean ± SE of four independent experiments.

Next, we explored other possible pathways activated in infected monocytes, which could account for the production of proinflammatory and regulatory cytokines. In particular, we focused on MAPK signalling that has been described to be involved in the production of inflammatory cytokines (such as TNF-*α*, IL-6, and IL-12p40) and therefore considered important for the initiation of an effective immune response against Mtb [14, 21–24]. Having found that Mtb induced rapidly both ERK phosphorylation and p38 phosphorylation [9], we investigated the involvement of these pathways on IL-23 expression given its key role in controlling the inflammatory response directed against Mtb [14, 25]. As control, we analyzed the effects induced by the addition of these MAPK inhibitors on the Mtb-induced expression of TNF-*α*, which has been previously demonstrated to be regulated by MAPK [22, 26]. Total RNA was extracted in monocyte cultures pretreated for 30 min with MAPK inhibitors and afterward infected with Mtb for additional 8 hr. We observed that p38 was likely involved in Mtb-induced IL-23 and TNF-*α* expression since a strong reduction of transcripts coding for TNF-*α* and IL-23p19 subunit was observed in Mtb-stimulated cultures pretreated with SB203580. On the contrary, the Mtb-stimulated expression of IL-23p19 was slightly reinforced while that of TNF-*α* was only partially reduced by the Erk inhibitor PD98059 (Figure 4(a)). On the other hand, the release of TNF-*α* and IL-23 was reduced when monocytes were stimulated with Mtb for 24 hr in presence of MAPK pharmacological inhibitors (Figure 4(b)). Taken together these results indicate that Mtb utilizes the MAPK signalling to promote the expression of the regulatory cytokine IL-23, which is in turn involved in the fine regulation of Th1/Th17 balance [16].

## 4. Conclusions

The importance of type-1 cytokines (IL-12, IL-23, and IFN-*γ*) in the regulation of innate and adaptive immunity against different intracellular pathogens, including Mtb, has been largely demonstrated both in animal models as well as in individuals with deficiencies in type-1 cytokine signalling pathways, which have an enhanced susceptibility to environmental mycobacteria or to the vaccine strain BCG [3, 14, 27]. The emerging picture from these findings indicates that the interplay between Mtb and phagocytes is crucial for the final outcome of Mtb infection.

Within this scenario, the role of monocytes has been underestimated since the majority of the analysis on the effect induced by Mtb infection in phagocytes has been performed in macrophages and DC. In this study, we have characterized the signalling pathways and the expression of regulatory and proinflammatory cytokines in human primary monocytes infected by Mtb. Interestingly, a selective expression of cytokines known to be involved in the establishment and maintenance of an inflammatory state was observed. Indeed, the simultaneous release of IL-23, IL-6, IL-1-*β*, and TNF-*α* and the lack of IL-12, IFN-*β*, and IL-27 production from infected monocytes, likely recruited in the lung, might impact on both innate and adaptive immune response directed against Mtb. It has been shown that IL-12 family cytokines are involved in the autocrine/paracrine regulation of antimycobacterial activity of macrophages [28, 29]. Indeed, the treatment with IL-12 and a soluble receptor for IL-27 during infection reduced the growth of bacteria recovered from macrophages. Moreover, IL-12 and IL-27 balance influenced also the profile of proinflammatory cytokines and chemokines produced by macrophages. In particular, the neutralization of IL-27 by soluble receptor has been shown to augment the levels of TNF-*α* and IFN-*γ* during early infection and may allow macrophages to better combat Mtb at a critical time. Based on these evidences, we can envisage that the absence of IL-27 production from Mtb-infected monocytes could lead to a more efficient elimination of mycobacteria, which is crucial for the containment of the infection.

In addition to the modulation of antimicrobial activity, the cytokines released by Mtb-infected monocytes may also influence the induction of T-cell immunity acting on the balance between Th1/Th17 memory responses. Indeed, IL-12p70 is required for the optimal IFN-*γ* T-cell response, which is crucial for control of Mtb growth while IL-23 can induce IFN-*γ* responses in the lung if IL-12 is not present. However, the major property of IL-23 is the ability to sustain the function of IL-17-producing *γδ* T [30] and the Th17 expansion [15, 16], which are, respectively, essential component of the early and late protective immune response directed against intracellular pathogens within the lung, respectively.

Although the role of Th17 in the immune response to Mtb is still controversial [31], future vaccine strategies have to consider also a correct stimulation of monocytes, which participate to the immune response against Mtb not only by supplying the infected lung tissues with precursors of macrophages and DC, but also providing a cytokine milieu favouring the IL-17 response.
